# Hospitalization Services Utilization Between Permanent and Migrant Females in Underdeveloped Rural Regions and Contributing Factors—A Five-Time Data Collection and Analysis

**DOI:** 10.3390/ijerph16183419

**Published:** 2019-09-14

**Authors:** Xiaotong Wen, Huilie Zheng, Zhenyi Feng, Winter Tucker, Yuanan Lu, Zhaokang Yuan

**Affiliations:** 1 Jiangxi Province Key Laboratory of Preventive Medicine, School of Public Health, Nanchang University, Nanchang 330006, China; 18070504083@163.com (X.W.); zhenghuilie@163.com (H.Z.); 2 Office of Public Health Studies, University of Hawaii at Mānoa, Honolulu, HI 96822, USA; fengzhenyi@shphschool.com; 3 School of Community Science, University of Nevada, Reno, NV 89557, USA; winter.nicolette@gmail.com

**Keywords:** migrant females, permanent females, hospitalization services utilization, underdeveloped rural regions

## Abstract

The proportion of migrating females has increased, and more often, old females are left in rural regions. Resources are needed to provide suitable hospitalization service to females in underdeveloped rural regions. Using multi-stage hierarchical cluster random sampling method, nine towns from three counties were enrolled in five-time points between 2006 and 2014 in this study. The research subjects of this study were females age 15 and up. Data regarding the utilization of inpatient services were collected and analyzed. Complex sampling logistic regression was conducted to analyze influencing factors. This study reveals that for both permanent females and migrant females, the older their age, the higher their hospitalization rate. The utilization of hospitalization service for permanent females was associated with the occurrence of chronic diseases (adjusted Odds Ratio (aOR) = 5.402). In addition, permanent females suffering from chronic diseases were more likely to avoid hospitalization despite their doctor’s advice (aOR = 34.657) or leave the hospital early against medical advice (AMA) (aOR = 10.009). Interventions to combat chronic diseases and adjust compensation schemes for permanent females need to be provided.

## 1. Introduction

Previous studies demonstrated that there are significant differences in access to and utilization of healthcare between urban and rural areas in China [1,2]. The healthcare system in rural areas is entirely different from the healthcare system in urban areas because of differences in social welfare; urban areas have a significantly better healthcare system than rural areas [3]. For example, the hospitalization rate of urban residents is always higher than rural residents from 2003 to 2013 according to the *China health statistics yearbook* 2018. The distance to the nearest medical facility for most of rural residents is farther than urban residents. Furthermore, 64.4% of rural residents’ access to healthcare is in a village clinic, for which service function is low. 25.8% of urban residents often go to general hospitals when they need to see a doctor [4]. The study revealed that inadequate access to healthcare was significantly higher among adults in rural areas than in urban areas (9.1% versus 5.4%; *p* < 0.01) [1].

Population mobility usually refers to the behavior of groups leaving their original place of residence and moving to a new place for reasons including seeking job opportunities, searching for educational resources, retirement, and getting married [5,6]. Internal migration underpins differences in population change and structure across subnational areas. Understanding how internal migration changes the population composition of local areas is critical for responding to healthcare needs [7,8].

In China, the migrating population is uniformly defined as people whose household registrations are not the same as their current residences, and have left their registered areas for six months or more [9]. In this way, a migrating population simply means that migrant people have changed their place of residence, but did not change their original household registration [10]. In China’s official discourse system, population migration refers to the cross-regional transfer of household registration management relationship approved by the household registration administration authority, whereas population mobility refers to a change of domicile without the transfer of household registration management. The migrating population is a concept developed under the conditions of China’s household registration (or Hukou) system, and is also a unique phenomenon [11]. China’s social welfare is closely tied to the status in Hukou system [6].

Economic development has led to a rising migrant population in China. Specifically, a large number of people move to eastern China for jobs and business because of inequality of economic development between eastern areas and central/western areas [12]. The ‘Report on the Development of China’s migrating Population’ 2018 showed that the scale of China’s migrating population was entering an adjustment period. Data from the ‘Report on the Development of China’s migrating Population’ 2017 showed that the scale of China’s migrating population in 2016 was 245 million. This represents a big change due to the adjustment of China’s industrial structure, the development of social economy, and the fact that the rural population in underdeveloped areas has flocked to developed, coastal cities to seek jobs over the past decade. It is estimated that the migrating population in China will gradually increase to 282 million in 2020, and up to 307 million by 2025, respectively. This number is expected to reach 327 million by 2030. According to the ‘Report on the Development of China’s migrating Population’ 2017, the proportion of females in China’s migrating population has increased, from 47.7% in 2011 to 48.3% in 2016. There are more migrating women in the 20 to 29 age group than men, but fewer females than males in other age groups. From 2011 to 2016, the sex ratio of floating population in China decreased from 109.6 in 2011 to 107.2 in 2016. Afterwards, a balance was maintained. 

The migrating population may have a higher risk of three main diseases in China: infectious diseases, maternal health and occupational diseases, and injuries [13]. Migrants face many obstacles in accessing essential health care services due to factors such as social welfare or healthcare system [14]. Additionally, it is possible that most of migrating population currently focus on their economic status, ignoring their own health [15]. Almost all the rural residents were covered under the New Rural Cooperative Medical Scheme in recent years, and this insurance has some effect on improving health service utilization in rural residents, especially permanent residents [16]. 

As a vulnerable group in society, women tend to have relatively poor physical fitness, and are also under dual pressure. They still have to take care of the family, while also considering the development of their careers. Additionally, inequality in education, employment, and income due to social discrimination against women results in an unequal access for women to get health services. They receive relatively low levels of attention due to the negative influence of the traditional “boy preference” in China. This inequality may reduce the chance in women to acquire the best health care. However, some researchers have confirmed that both migrant females (male’s OR = 0.74, CI: 0.58–0.95) and permanent females (male’s OR = 0.78, CI: 0.69–0.89) had higher health needs [14]. For a long time, rural women’s demand for medical treatment cannot be fully satisfied, due to the low socioeconomic status. One recent study showed that significant gender inequalities in current health care service utilization exist [17]. Compared to males, females have a worse perception of health status and higher non-communicable diseases (NCD) rates [17]. Gender inequalities still exist in Jiangxi in terms of the demand for and utilization of health care services [18]. 

Meanwhile, Jiangxi Province has undergone major changes over its rural population structure and quantity due to population mobility in the last few years. Migrant people are mostly young males, and the residents staying in rural areas are mainly women, children, and the elderly. Females have become a major component of the permanent resident population. This represents a typical example of an underdeveloped region. 

This study compared the current status of females’ hospitalization between permanent and migrant female residents, and analyzed the influencing factors relating to the hospitalization due to illnesses. Provide some insights into the formulation and adjustment of health policies in order to improve women’s health service utilization.

## 2. Materials and Methods

### 2.1. Data Collection

This survey was initiated in Jiangxi Province in 2006 and continued every other year (2008, 2010, 2012, and 2014), using the multistage stratified random cluster sampling method. First, according to the basis of the sampling method from the Ministry of Health of the People’s Republic of China, all the counties of Jiangxi Province were ranked based on farmers’ average incomes. These counties were classified into three groups by the percentile method (<33.33%, 33.33–66.67% and >66.67%). Next, three counties (Xiushui, Wuyuan, and Luxi) were selected, and each represented one of the three economic levels. The percentile method was employed to choose three towns from each county. Similarly, three administrative villages were enrolled from each of the nine towns. Finally, all households from the sampled villages were sorted by house numbers, with the first house number being randomly determined. All the family members from 70 households were investigated successively as described previously [19].

In this survey study, a one-to-one, home-visiting investigation method was employed. By asking homeowners questions, trained investigators completed the questionnaires for the participants. If the homeowner was out, the questions were answered by other family members age 18 or older. The survey questions included (a) general demographic characteristics, (b) health status of family members, (c) the needs and utilization status of health care services of family members, and (d) health expenses and reimbursement status [20]. This study was a retrospective cross-sectional investigation, which was approved by the Medical Ethics Committee of Nanchang University and performed in accordance with local ethical guidelines. Participants consent was verbal. The research subjects of this study were females age 15 and up, subjects were divided into two groups: permanent female residents (those who have been living in local villages for more than half a year) and female migrants (those who have a locally registered household, but have gone out to work or study during the past six months, including their companions). 

### 2.2. Calculation of Sample Size

Sample size estimation used the formula:

$$n=\frac{\left[Z_{\alpha /2}^{2}*p\left(1-p\right)\right]}{\delta^{2}}$$

where *n* is the sample size and *p* is the hospitalization rate for base-line survey (*p* ≈ 5%) and *Z_α_*_/2_ is normal deviation for a two-tailed alternative hypothesis. The level of significance *α* is set at 0.05, so *Z*_0.05/2_ = 1.96. *δ* is the desired level of margin of error (usually 0.01). According to these criteria, the sample was calculated as *n* = 1825. This research also used a cluster sampling method, making it better to investigate by plus 0.5*n*. Finally, a minimal sample size for cluster sampling was calculated as *n*_1_, thus *n*_1_= *n* + 0.5*n* = 2737.5 ≈ 2738, which is far below the actual sample size (7500~) for the total length of the study. 

### 2.3. Weighting Method

Complex sampling surveying is a method of extracting the research subjects randomly for each group according to required proportions, after dividing subjects into different groups. Considering the possible presence of sampling errors, there are obvious deviations between the overall population and the samples, which leads to differences in the results of the investigation compared with the overall characteristics. Therefore, hierarchical weighting was measured afterwards to correct the data and to reduce the error. The calculation of the weight consists of two parts: individual basis weight and adjustment weight and their product that makes the final weight of the individual [21,22]. The following shows the data weighting method in this study.

This study used a three-stage sampling method. Suppose the sampling weight of the stage 1 sampling unit was *W*_1_, that of the stage 2 sampling unit was *W*_2|1_, and that of the stage 3 sampling unit was *W*_3|2,1_. Individual basis weight (*W_b_*) was the product of the sampling weights of the three stages: *W_b_* = *W*_1_ × *W*_2|1_ × *W*_3|2,1_.

Subjects’ genders were stratified into two groups, with r = 1,2; the ages were stratified into eight groups, with c = 1,2,3,4,5,6,7,8. As a result, there were a total of 16 groups (2 × 8 = 16). The calculation formula for adjustment weight is as below:

$$W_{\mathrm{adj}}=\frac{N_{\mathrm{rc}}}{\sum_{i=1}^{N_{\mathrm{rc}}}W_{i}}$$

where *N*_rc_ is the total number of people corresponding to the number of r groups of gender and number of c groups of age in the 16 groups. It is the sum of the basis weights of all the individuals who are subjects in that group. The final weight of the individual is the product of basis weight and adjustment weight, and its formula was:

$$W_{f}=W_{b}\times W_{\mathrm{adj}}=W_{1}\times W_{2|1}\times W_{3|2,1}\times W_{\mathrm{adj}}=W_{1}\times W_{2|1}\times W_{3|2,1}\times \frac{N_{\mathrm{rc}}}{\sum_{i=1}^{N_{\mathrm{rc}}}W_{i}}$$

### 2.4. Indexes Construction

The hospitalization rate means the proportion of females hospitalized due to illness compared to the total number of females surveyed in the past year (%). Hospitalization was measured by asking whether respondents received any hospitalization services in the past year. The rate of female hospital avoidance means the proportion of females who should be receiving hospitalized services but were not, compared to those who should have been hospitalized in the past year and were, in fact, hospitalized (%). The rate of females who left the hospital early against medical advice (AMA) refers to the proportion of females who left the hospital early against medical advice compared to the number of hospitalizations in the past year. For the levels of income, 0–3000 RMB annual per capita is considered low, 3001–6000 RMB annual per capita is considered middle, and 6001 and above RMB annual per capita is considered high.

### 2.5. Quality Control

To ensure comparability of the data, the sample villages and towns selected for tracking investigation and baseline survey were all the same. The investigators were graduate students from the Department of Public Health, Nanchang University with experience in survey study. They received training together and knew the standard language before conducting the survey. During the investigation, they worked with village cadres who helped with needed translation. At the end of the investigation, all the investigators gathered to import the data. If there were any mistakes or unfilled blanks on the questionnaires, respondents or local village cadres were contacted in a timely manner, and questions were asked of research subjects through phone calls, to complete the questionnaire. For the same questionnaire, two people input data on two computers to check for logical errors and other deficiencies. 

In this study, the Myer’s blended index was used to evaluate the quality of the survey data [23]. It was assumed that in a population without any data preferences, the age group ending in any of the digits 0–9 should account for one-tenth of the population. The absolute value of the difference between the actual population age distribution and the theoretical distribution is called the Myer’s blended index. The Myer’s blended index ranges from 0 to 99.0, which means that the implemented data strictly conform to the theoretical distribution, and there are no accumulation phenomenon. A value of 99 refers to the upper age of the population. In general, due to the phenomenon of death and migration in all age groups and the inconsistency of death probability migration rate in all age groups, the actual population age distribution is deviated from the theoretical distribution. However, the Myer’s blended index cannot be larger than 60 indicating that there is a serious age preference, which was named an accumulation phenomenon in this survey population data. The Myer’s blended index less than 60 suggests an overall good data quality. The Myer’s blended indexes of five surveys in this study were 5.12, 12.02, 8.52, 3.54, and 7.07, respectively. The calculation process is shown in the Table 1.

### 2.6. Data Analysis

Epidata 3.0 was used to input the data, and the database was imported into Excel. The database was transferred into SPSS 24.0 for statistical analysis. For the comparison and categorization of inter-individual differences, the χ^2^ testing statistical method was applied after complex sampling weighting. Complex sampling logistic regression was used for analysis and adjusted Odds Ratio (aOR) was calculated by multivariate logistic regression [24]. The significance level was α = 0.05.

## 3. Results

Table 2 shows that the percentage of females aged 15–34 was 43.6% while the aged group ≥55 was 20.0% (the lowest percentage). Total unmarried females accounted for 19.4%, married females covered the highest percentage (73.8%). The percentage of literacy was 41.2% and females with middle level income accounted for 54.0%, as compared to 20.8% for those with high level of income. Chronic disease prevalence among all female subjects was 11.2%, while migrant females had a percentage of 27.8.

As shown in Table 2, most migrant females (78.3%) were in the age group of 15–34 years and less than 2% were ≥55 years old, while permanent females aged 55 and over accounted for 27.0%. The majority of migrant females were non-farmers (87.8%), whereas the majority of permanent females were farmers (70.6%). The unmarried migrant females accounted for 36.9%, which was higher than that in permanent females (12.6%). Nearly 80.0% of migrant females had a junior high school and above education background as compared to that of 50.6% of permanent females. Migrant females at the high-income level comprised 24.8% of the population, which was significantly higher than the 19.2% determined for permanent females. More than 85.9% of migrant females had a job as compared to 70.1% in permanent female group. The chronic disease rate in migrant females was 2.8%, which is significantly lower than the 14.5% reported for the permanent females (*p* < 0.001). This study also revealed statistically significant differences between migrant females and permanent females in terms of their age, career status, marital status, education level, income level, labor force, and status of chronic diseases (*p* < 0.05).

The hospitalization rate increased for all females from 2.7% in 2006 to 6.7% in 2014, except for a slight decrease in 2010 (Table 3). Among all females including permanent and migrant groups, the hospitalization rate is linked with their age: the older the age, the higher the rate. For female farmers, the hospitalization rate was higher than that of non-farmers (2.8%); females with a lower education level experienced a higher hospitalization rate (7.5%) in comparison to those with a higher education level. The hospitalization rate for married females was 5.4%, which was much lower than that detected in divorced or widowed females. Non-working females experienced a higher hospitalization rate (8.0%) than working ones. As shown in Table 3, females with chronic diseases had a significantly higher hospitalization rate than those without the diseases (*p* < 0.05). Statistical uncertainties are present in all of the indicators above (*p* < 0.05).

The hospitalization rate for permanent female residents was 5.9%, which was higher than that for migrant females (2.0%). This trend kept the same for the years of the five-point time study. Similarly, the hospitalization rate for permanent female residents was higher than that for migrant females between farmers and non-farmers. Among the respondents with different education and income level, the hospitalization rate of permanent female residents was significantly higher than that of migrant females. However, the hospitalization rate for the aged group ≥55 was very similar between migrant females (11.6%) and permanent female residents (11.2%).

Complex sampling logistic regression analysis shows that the hospitalization rate of migrant females significantly increased from 2006 to 2014 (Table 4). This survey indicates that it was 2.011 and 2.860 times more likely, respectively, for migrant females aged 35–54 and ≥ 55 to be hospitalized than those in the 15–34 age groups. Compared to divorced or widowed migrant females, married women were 1.652 times more likely to be hospitalized. The logistic regression analysis also showed that the risk for hospitalization for females with chronic diseases was 2.996 times higher than that of the females suffering no chronic diseases (Table 4).

Complex sampling logistic regression analysis shows that the hospitalization rate of permanent females significantly increased from 2006 to 2014 (Table 4). This survey indicates that it was 2.803 and 7.526 times more likely, respectively, for permanent females aged 35–54 and ≥ 55 to be hospitalized than those in the 15–34 age groups. The logistic regression analysis also showed that the risk for hospitalization for females with chronic diseases was 5.402 times higher than that of the females suffering no chronic diseases (Table 4).

Complex sampling logistic regression analysis shows that the hospital avoidance of migrant females significantly increased from 2006 to 2012 (Table 5). This survey indicates that there were 3.745 and 2.424 times more likely for migrant females aged 35–54 and ≥55 to avoid hospitalization compared to those in the age groups of 15–34. This survey indicates that it was 2.681 times more likely for farmers to avoid hospitalization compared to non-farmers. The logistic regression analysis also showed that the risk for hospital avoidance for migrant females with chronic diseases was 21.070 times higher than that of the females suffering no chronic diseases (Table 5).

The survey for permanent females indicates that it was 3.220 times more likely for permanent females aged ≥55 to be avoid hospitalization compared to permanent females in the age groups of 15–34. Furthermore, this survey indicates that it was 1.542 times more likely for farmers to avoid hospitalization compared to non-farmers. Additionally, this survey indicates that it was 0.421 times more likely for working groups to avoid hospitalization compared to non-working groups. The logistic regression analysis also showed that the risk for hospitalization for females with chronic diseases was 34.657 times higher than that of females suffering no chronic diseases (Table 5).

As shown in Table 6, complex sampling logistic regression analysis revealed that it was 4.729 times more likely for migrant females aged 35–54 to be leaving the hospital early and against medical advice compared to females age 15–34. 

As shown in Table 6, complex sampling logistic regression analysis revealed that there were 8.687 times and 2.007 times more likely, respectively, for permanent females age 35–54 and ≥55 to be early leaving hospital against medical advice compared to females age 15–34. The risk for early leaving hospital against medical advice among migrant females with chronic diseases was 10.009 times higher than that of the females who did not have the chronic diseases. 

## 4. Discussion

As a large labor-power exporting province, Jiangxi has undergone important changes in terms of its population structure and quantity. Shortcomings of the present medical resource allocation system based on the registered population have been encountered. The consequences of this situation may lead to a short of or ineffective medical resource to meet the medical need [25]. Previous studies revealed that some demographic factors might impact on the hospital health services utilization, where females were more likely to use health service [26]. Females are known to be more sensitive to and have a higher awareness of health problems and symptoms, while males are more inclined to self-sustain and self-medicate when they felt unwell [27,28]. Our findings provide scientific baseline information for the improvement of current hospitalization services for females in underdeveloped rural areas.

A recent report from Italy has indicated that the hospitalization rate for females was 6.8%, which is consistent with the results of this study [17]. This study reveals that among all female population, the older their age, the higher their hospitalization rate, which was consistent with the findings from previous study [29]. One possible explanation for this may be that endocrine regulation experiences changes when people grow older and body conditions start to worsen, which triggers more diseases [30]. The higher the education level, the lower the hospitalization rate; married females had a lower rate than unmarried, divorced, or widowed females. A previous study conducted in rural China reported low utilization of health services among left-behind elderly and married females. The risk of illness and severity of disease were also higher than that of non-left-behind elderly [31]. Further analysis revealed that those females with lower education are mostly older, and they often have a relatively worse living standard and pay less attention to their health conditions. Living conditions of married females are quite different from divorced or widowed in several aspects including their experience in the lack of family support and income level. This study revealed that working female groups had a lower hospitalization rate than those non-working ones. Presenteeism among working groups may be a reason because they simply need the money and cannot afford to take time off due to illness. 

The utilization of hospitalization service for female was also associated with the prevalence of chronic diseases (aOR = 3.098); thus, females with chronic diseases had a higher hospitalization rate than those without the diseases. Our study also showed that the female suffering from chronic diseases were more likely to avoid being hospitalized even though their doctor advised treatment (aOR = 25.766) and more likely to leave hospital early against medical advice (aOR = 5.118). The current utilization of inpatient medical services for females with chronic diseases was not well received. Other studies showed that the weaknesses of the primary healthcare are one of the major causes of difficulties and high expenses in medical care [3,32]. If females with chronic diseases have access to adequate and timely primary care or outpatient services, hospitalization may reduce [33].

Given than the young age and overall good health outcomes, the hospitalization rate due to illnesses of migrant females (2.0%) is lower than the permanent females (5.9%). One possible cause for this finding is that most of the permanent females were over 35 or older and their general health was not as good as migrant young females [13]. Additionally, it is also possible that most of migrant females currently focus on their economic status, ignoring their own health status. The migrant females were younger than permanent females, and less than 2% of the migrant females were ≥55 years old, thus weakening the effect of age on health needs. Between farmer and non-farmer migrant females, the former group had a lower hospitalization rate. On the contrary, the hospitalization rate of farmers was higher than that of non-farmers in permanent females. These results may be due to the difference in distribution of career status between permanent and migrant females. 

Due to the fact of lower education and lower income, permanent females also had lower standards of living condition. It was detected that in order to save money, permanent females are more likely to conduct “disease diagnosis by themselves” when they have minor illnesses, which could make them vulnerable to “more serious illnesses” and be hospitalized. Since their husbands are migrant workers away from home, permanent females have to stay home alone, encountering weaker mental health than migrant females who live with their husband. The medical services located in the rural areas are nearby, and thus, permanent females could have much easy access to medical care with less service fee [8]. However, permanent females’ access to healthcare in village clinics or the township health centers which service function are low. The current situation argues for the allocation of more high quality medical resources for inpatient facilities in rural town hospitals where the rural female residents are treated. Particularly, more resources should be added for the treatment of the diseases that the elderly are more susceptible to (i.e., cardiovascular and cerebrovascular diseases [34]). Furthermore, the government ought to introduce adequate and experienced health workers in remote and economically underdeveloped provinces by giving extra subsidies and other preferential policies to ameliorate the inequality status of health worker [3,5].

With the rapid movement of population, the population composition of rural permanent residents has undergone major changes. The orientation of medical resources and the rationality of the layout of medical institutions at all levels have become the focus of attention. Female health care has been listed on the top priority in China medical care system today. More high quality medical resource for permanent females needs to be provided in order to adequately combat the inpatient diseases of the elderly.

In order to improve the situation of utilization of essential hospitalization services, it is necessary to develop and implement a more comprehensive approach for the prevention and treatment of chronic diseases for the entire population. Future studies are needed to lay more emphasis on disease analysis, to figure out the determinant factors to those females who are unwilling to seek medical care, including economic constraints, and to facilitate an even distribution and utilization of medical resources in the future.

### Limitations

The data of this research was based on the multi-stage stratified cluster sampling as planned, investigation was conducted with the assistance of local government department staff and village cadres, and the data of each household was collected through interviewing homeowners. Using this method, bias caused by nonresponse was avoided, as all the families answered the questions on the questionnaire; however, there might have been selection bias caused by the assistance of the officials. Therefore, an afford was maintained during the survey to keep the local government department staff and village cadres from direct interactions with families as much as possible, thus, the trainer investigators were the ones to directly talk to the respondents. In this way, the data collected would be relatively accurate.

In this paper, there was only an overall description of the rate of female hospitalization due to illnesses; however, no analysis was performed regarding the disease composition. Thus, there is a need for future studies to focus on analyzing disease composition, as a better understanding of current hospitalization would be more informative for the governments to allocate their medical resources specifically in response to various diseases. The research subjects were females aged 15 and over only and the research lacked data from the male population, which makes it impossible to conduct a complete data comparison and create a better understanding of the general hospitalization rate in the region.

## 5. Conclusions

Permanent female residents in rural regions experienced a higher rate of hospitalization due to their illnesses as compared to migrant female population. The findings from this study suggest that the allocation of medical resources should be readjusted according to changes in the permanent resident population.

## Figures and Tables

**Table 1:** Myers blended index evaluation schedule of three counties in Jiangxi province in 2014.

The Ending Figure of Age	10–49 Years Old			20–59 Years Old				Percentage (%) (8)/45039	The Absolute Value of the Ninth Line Minus 10
	Population	Weight	(2) * (3)	Population	Weight	(5) × (6)	(4) + (7)		
(1)	(2)	(3)	(4)	(5)	(6)	(7)	(8)	(9)	(10) ^a^
0	390	1	390	419	9	3771	4161	9.24	0.76
1	377	2	754	426	8	3408	4162	9.24	0.76
2	424	3	1272	456	7	3192	4464	9.91	0.09
3	376	4	1504	361	6	2166	3670	8.15	1.85
4	444	5	2220	482	5	2410	4630	10.28	0.28
5	518	6	3108	530	4	2120	5228	11.61	1.61
6	461	7	3227	480	3	1440	4667	10.36	0.36
7	464	8	3712	528	2	1056	4768	10.59	0.59
8	478	9	4302	517	1	517	4819	10.70	0.70
9	447	10	4470	477	0	0	4470	9.92	0.08
							45,039	100.00	7.07

^a^: The absolute value from 10%.

**Table 2:** Distribution of demographic characteristics in three counties among total amount of females, migrant females, and permanent females. (%, 95% CI) *.

	Total Females	Migrant Females	Permanent Females	χ^2^	*p*
Number of respondents					
People surveyed	15,600	3972	11,628		
Weighted number	2,245,284	624,257	1,621,027		
Year					
2006	24.9 (10.9, 47.5)	28.7 (11.5, 55.5)	23.5 (10.6, 44.3)		
2008	19.3 (9.7, 34.8)	17.6 (8.1, 34.1)	19.9 (10.2, 35.2)		
2010	19.3 (7.3, 42.1)	17.1 (6.0, 39.9)	20.2 (7.8, 43.1)		
2012	18.9 (9.7, 33.8)	16.9 (7.7, 33.1)	19.7 (10.4, 34.2)		
2014	17.5 (8.8, 31.9)	19.7 (8.9, 38.1)	16.7 (8.7, 29.7)	4.367	0.011
Age					
15~	43.6 (41.0, 46.1)	78.3 (74.4, 81.8)	30.2 (27.7, 32.7)		
35~	36.4 (35.4, 37.5)	19.9 (17.2, 22.8)	42.8 (41.6, 44.0)		
55~	20.0 (18.2, 21.9)	1.8 (0.8, 3.9)	27.0 (24.9, 29.3)	260.438	<0.001
Career status					
Farmer	54.4 (47.7, 60.9)	12.2 (9.8, 15.1)	70.6 (61.5, 78.3)		
Non-farmer	45.6 (39.1, 52.3)	87.8 (84.9, 90.2)	29.4 (21.7, 38.5)	309.545	<0.001
Marital status					
Unmarried	19.4 (15.8, 23.4)	36.9 (31.9, 42.2)	12.6 (9.4, 16.7)		
Married	73.8 (70.5, 76.8)	61.6 (56.6, 66.2)	78.5 (75.4, 81.3)		
Divorced or widowed	6.8 (6.1, 7.8)	1.5 (0.9, 2.7)	8.9 (8.0, 10.0)	123.015	<0.001
Education level					
Elementary school	41.2 (37.1, 45.5)	20.1 (18.4, 21.9)	49.4 (44.1, 54.6)		
≥Junior high school	58.8 (54.5, 62.9)	79.9 (78.1, 81.6)	50.6 (45.4, 55.9)	445.106	<0.001
Income level					
Low	25.2 (15.3, 38.8)	21.7 (10.6, 39.2)	26.6 (17.2, 38.8)		
Middle	54.0 (50.1, 57.8)	53.5 (48.0, 58.9)	54.2 (50.4, 57.9)		
High	20.8 (12.9, 31.8)	24.8 (14.2, 39.7)	19.2 (12.3, 28.7)	5.542	0.018
Labor force					
Yes	74.5 (72.1, 76.7)	85.9 (83.3, 88.1)	70.1 (67.6, 72.4)		
No	25.5 (23.3, 27.9)	14.1 (11.9, 16.7)	29.9 (27.6, 32.4)	276.864	<0.001
Chronic diseases					
Yes	11.2 (10.3, 12.2)	2.8 (2.1, 3.7)	14.5 (13.3, 15.7)		
No	88.8 (87.8, 89.7)	97.2 (96.3, 97.9)	85.5 (84.3, 86.7)	309.215	<0.001
Migrant					
Yes	27.8 (26.6, 29.0)				
No	72.2 (71.0, 73.4)				

* Pearson Chi-Square test between migrant females and permanent females. CI: confidence interval.

**Table 3:** The hospitalization rate due to illnesses of female among total females, migrant females, and permanent females (%) *.

Demographic characteristics	Total Females	Migrant Females	Permanent Females	χ^2^	*p*
Year					
2006	2.7	3.3	1.4	2.417	0.171
2008	4.4	5.3	1.8	7.561	0.033
2010	3.7	4.5	1.5	4.716	0.073
2012	5.8	7.3	1.6	78.114	<0.001
2014	6.7	8.5	3.0	63.859	<0.001
χ^2^	5.612	7.447	1.105		
*p*	0.021	0.005	0.335		
Age					
15~	1.5	1.7	1.2	1.358	0.267
35~	5.3	5.5	4.0	1.230	0.289
55~	11.2	11.2	11.6	0.008	0.931
χ^2^	81.524	47.558	18.724		
*p*	<0.001	<0.001	<0.001		
Career status					
Farmer	2.8	3.8	2.0	10.066	0.008
Non-farmer	6.1	6.6	1.9	17.005	0.001
χ^2^	27.575	10.467	0.037		
*p*	<0.001	0.007	0.851		
Marital status					
Unmarried	5.4	6.4	2.4	16.868	0.001
Married	1.2	1.2	1.2	<0.001	0.984
Divorced or widowed	8.5	8.8	4.1	1.159	0.303
χ^2^	38.576	15.428	2.086		
*p*	<0.001	<0.001	0.158		
Education level					
Elementary school	2.9	3.6	1.7	14.068	0.003
≥Junior high school	7.5	8.3	2.9	14.119	0.003
χ^2^	118.982	94.690	2.878		
*p*	<0.001	<0.001	0.116		
Income level					
Low	4.5	5.4	1.6	16.666	0.002
Middle	4.5	5.5	1.7	18.102	0.001
High	6.1	7.8	2.7	21.916	0.001
χ^2^	2.418	4.320	0.795		
*p*	0.119	0.030	0.429		
Labor force					
Yes	3.7	4.6	1.9	21.893	0.001
No	8.0	9.1	2.2	47.376	<0.001
χ^2^	52.644	51.187	0.153		
*p*	<0.001	<0.001	0.703		
Chronic diseases					
Yes	15.9	16.2	12.2	27.015	<0.001
No	3.4	4.2	1.7	1.208	0.293
χ^2^	378.394	307.702	109.593		
*p*	<0.001	<0.001	<0.001		
Migrant					
Yes	2.0				
No	5.9				
χ^2^	41.986				
*p*	<0.001				

* Pearson Chi-Square test between migrant females and permanent females.

**Table 4:** The analysis results of hospitalization due to illnesses using complex sampling logistic regression of females aged 15 and over among total, migrant, and permanent females. aOR: adjusted Odds Ratio.

	Total Females aOR (95%CI)	Migrant Females aOR (95%CI)	Permanent Females aOR (95%CI)
Year			
2006	1	1	1
2008	1.503 (0.958, 2.357)	1.039 (0.644, 1.677)	0.818 (0.338, 1.980)
2010	1.284 (0.647, 2.549)	1.197 (0.584, 1.631)	1.169 (0.883, 1.346)
2012	2.336 (1.637, 3.332) *	1.360 (0.814, 1.956)	1.039 (0.871, 3.985)
2014	2.299 (1.154, 4.581) *	1.412 (1.228, 1.744) *	1.910 (1.186, 4.446) *
Age			
15~	1	1	1
35~	2.246 (1.523, 3.313) *	2.011 (1.110, 3.644) *	2.803 (1.432, 5.488) *
55~	3.296 (2.087, 5.208) *	2.860 (1.543, 5.303) *	7.526 (2.178, 26.008) *
Career status			
Non-farmer	1	1	1
Farmer	1.101 (0.802, 1.512)	1.171 (0.761, 1.801)	0.413 (0.191, 0.893) *
Marital status			
Unmarried	1	1	1
Married	0.875 (0.531, 1.439)	0.669 (0.276, 1.625)	0.911 (0.171, 4.856)
Divorced or widowed	1.634 (1.141, 2.340) *	1.652 (1.151, 2.370) *	1.107 (0.325, 3.773)
Education level			
≤elementary school	1	1	1
≥Junior high school	0.789 (0.638, 0.976) *	0.764 (0.610, 0.957) *	1.059 (0.553, 2.029)
Income level			
High	1	1	1
Middle	0.847 (0.627, 1.143)	0.850 (0.676, 1.069)	0.747 (0.259, 2.155)
Low	0.819 (0.610, 1.089)	0.824 (0.614, 1.104)	0.695 (0.215, 2.243)
Labor force			
No	1	1	1
Yes	0.514 (0.380, 0.697) *	0.490 (0.372, 0.645) *	0.795 (0.370, 1.709)
Chronic diseases			
No	1	1	1
Yes	3.098 (2.630, 3.650) *	2.996 (2.550, 3.521) *	5.402 (2.592, 11.260) *

* p < 0.05; aOR: adjusted Odds Ratio; CI: confidence interval.

**Table 5:** The analysis results of hospital avoidance using complex sampling Logistic regression of females aged 15 and over among total females, migrant females, and permanent females.

	Total Females aOR (95%CI)	Migrant Females aOR (95%CI)	Permanent Females aOR (95%CI)
Year			
2006	1	1	1
2008	0.167 (0.051, 0.548) *	0.162 (0.048, 0.547)	0.962 (0.838, 1.138)
2010	0.918 (0.457, 1.845)	1.179 (0.532, 2.614)	0.320 (0.068, 1.507)
2012	1.854 (0.968, 3.551)	2.128 (1.147, 3.945) *	1.751 (0.968, 2.750)
2014	1.491 (0.707, 3.142)	1.472 (0.671, 3.229)	1.161 (0.707, 2.143)
Age			
15~	1	1	1
35~	3.621 (1.563, 8.387) *	3.745 (1.818, 7.716) *	12.687 (0.791, 23.472)
55~	2.451 (0.987, 6.090)	2.424 (1.188, 4.945) *	3.220 (2.988, 6.579) *
Career status			
Non-farmer	1	1	1
Farmer	1.844 (1.246, 2.728) *	2.681 (1.369, 5.251) *	1.542 (1.367, 2.802) *
Marital status			
Unmarried	1	1	1
Married	2.270 (1.060, 4.862) *	1.875 (0.581, 6.058)	2.270 (1.062, 4.853) *
Divorced or widowed	0.638 (0.409, 0.994) *	0.603 (0.352, 1.033)	0.839 (0.629, 1.697)
Education level			
≤elementary school	1	1	1
≥Junior high school	0.777 (0.422, 1.432)	0.743 (0.403, 1.372)	0.912 (0.605, 3.862)
Income level			
High	1	1	1
Middle	1.039 (0.628, 1.718)	0.934 (0.530, 1.645)	1.896 (0.827, 2.313)
Low	1.699 (0.828, 3.487)	1.518 (0.734, 3.140)	2.579 (0.936, 4.357)
Labor force			
No	1	1	1
Yes	0.551 (0.325, 0.934) *	0.644 (0.333, 1.246)	0.421 (0.325, 0.834) *
Chronic diseases			
No	1	1	1
Yes	25.766 (8.983, 73.906) *	21.070 (8.887, 49.956) *	34.657 (19.634, 56.852) *

* p < 0.05; aOR: adjusted Odds Ratio; CI: confidence interval.

**Table 6:** The analysis results of the early leaving hospital against medical advice using complex sampling Logistic regression of females aged 15 and over among total females, migrant females, and permanent females.

	Total Females aOR (95%CI)	Migrant Females aOR (95%CI)	Permanent Females aOR (95%CI)
Year			
2006	1	1	1
2008	0.363 (0.153, 0.858)	0.433 (0.159, 1.183)	0.352 (0.063, 1.423)
2010	0.549 (0.253, 1.194)	1.108 (0.477, 2.569)	1.271 (0.717, 2.253)
2012	0.652 (0.211, 2.017)	1.013 (0.245, 4.197)	1.595 (0.585, 2.394)
2014	1.203 (0.413, 3.503)	2.842 (0.736, 10.970)	1.505 (0.046, 49.710)
Age			
15~	1	1	1
35~	2.939 (1.069, 8.080) *	4.729 (1.398, 16.003) *	8.687 (1.077, 70.094) *
55~	2.648 (0.913, 7.677)	2.906 (0.913, 9.252)	2.007 (1.724, 2.335) *
Career status			
Non-farmer	1	1	1
Farmer	3.276 (1.310, 8.193) *	2.948 (0.549, 15.842)	3.186 (0.405, 25.069)
Marital status			
Unmarried	1	1	1
Married	0.828 (0.054, 12.665)	0.643 (0.096, 4.279)	1.756 (0.086, 19.853)
Divorced or widowed	1.158 (0.441, 3.039)	0.679 (0.242, 1.904)	0.892 (0.539, 2.008)
Education level			
≤elementary school	1	1	1
≥Junior high school	1.285 (0.642, 2.573)	1.275 (0.586, 2.775)	0.933 (0.070, 12.374)
Income level			
High	1	1	1
Middle	0.956 (0.478, 1.913)	1.152 (0.586, 2.264)	1.240 (0.137, 11.225)
Low	0.643 (0.273, 1.515)	0.904 (0.306, 2.668)	0.044 (0.001, 1.410)
Labor force			
No	1	1	1
Yes	0.785 (0.291, 2.116)	1.419 (0.515, 3.909)	3.039 (0.342, 7.042)
Chronic diseases			
No	1	1	1
Yes	5.118 (2.134, 12.278) *	2.313 (0.850, 6.296)	10.009 (3.076, 13.704) *

* p < 0.05; aOR: adjusted Odds Ratio; CI: confidence interval.

## References

[1] Zhang X., Dupre M.E., Qiu L., Zhou W., Zhao Y., Gu D. (2017). Urban-rural differences in the association between access to healthcare and health outcomes among older adults in China. BMC Geriatr..

[2] Zhai S., Wang P., Dong Q., Ren X., Cai J., Coyte P.C. (2017). A study on the equality and benefit of China’s national health care system. Int. J. Equity Health.

[3] Liu X., Li N., Liu C., Ren X., Liu D., Gao B., Liu Y. (2016). Urban-rural disparity in utilization of preventive care services in China. Medicine.

[4] National Health Commission (2018). China Health Statistics Yearbook 2018.

[5] Walls H.L., Vearey J., Modisenyane M., Chetty-Makkan C.M., Charalambous S., Smith R.D., Hanefeld J. (2015). Understanding healthcare and population mobility in southern Africa: The case of South Africa. S. Afr. Med. J..

[6] Mou J., Griffiths S.M., Fong H., Dawes M.G. (2013). Health of China’s rural-urban migrants and their families: A review of literature from 2000 to 2012. Br. Med. Bull..

[7] Rodriguez-Vignoli J., Rowe F. (2018). How is internal migration reshaping metropolitan populations in Latin America? A new method and new evidence. Popul. Stud..

[8] Peng Y., Chang W., Zhou H., Hu H., Liang W. (2010). Factors associated with health-seeking behavior among migrant workers in Beijing, China. BMC Health Serv. Res..

[9] Zhao Y., Kang B., Liu Y., Li Y., Shi G., Shen T., Jiang Y., Zhang M., Zhou M., Wang L. (2014). Health insurance coverage and its impact on medical cost: Observations from the floating population in China. PLoS ONE.

[10] Skeldon R. (1985). Population pressure, mobility, and socio-economic change in mountainous environments: Regions of refuge in comparative perspective. Mt. Res. Dev..

[11] Goldstein A., Goldstein S. (1988). Varieties of population mobility in relation to development in China. Stud. Comp. Int. Dev..

[12] Fang H., Yang L., Zhang H., Li C., Wen L., Sun L., Hanson K., Meng Q. (2017). Strengthening health system to improve immunization for migrants in China. Int. J. Equity Health.

[13] Hu X., Cook S., Salazar M.A. (2008). Internal migration and health in China. Lancet.

[14] Zheng L., Hu R., Dong Z., Hao Y. (2018). Comparing the needs and utilization of health services between urban residents and rural-to-urban migrants in China from 2012 to 2016. BMC Health Serv. Res..

[15] Lu L., Zou G., Zeng Z., Han L., Guo Y., Ling L. (2014). Health-related quality of life and its correlates among Chinese migrants in small- and medium-sized enterprises in two cities of Guangdong. PLoS ONE.

[16] Chen J., Yu H., Dong H. (2016). Effect of the new rural cooperative medical system on farmers’ medical service needs and utilization in Ningbo, China. BMC Health Serv. Res..

[17] Waure C.D., Bruno S., Furia G., Sciullo L.D., Carovillano S., Specchia M.L., Geraci S., Ricciardi W. (2015). Health inequalities: An analysis of hospitalizations with respect to migrant status, gender and geographical area. BMC Int. Health Hum. Rights.

[18] Tayeb S.E., Abdalla S., Bergh G.V.D., Heuch I. (2015). Use of healthcare services by injured people in Khartoum State, Sudan. Int. Health.

[19] Zhang L., Yuan Z., Maddock J.E., Zou J., Zheng Z., Zhou W., Zheng H. (2014). Chronic disease prevalence and influencing factors among rural residents in Jiangxi, China. Int. Health.

[20] Zou J., Yang W., Cook D.M., Yuan Z., Zhang L., Wang X. (2016). New cooperative medical financing policy and hospitalization in rural China: Multi-stage cross-sectional surveys. Int. Health.

[21] Pan B., Yuan Z., Zou J., Cook D.M., Yang W. (2016). Elderly hospitalization and the New-type Rural Cooperative Medical Scheme (NCMS) in China: Multi-stage cross-sectional surveys of Jiangxi province. BMC Health Serv. Res..

[22] Platt R.W., Harper S.B. (2013). Survey data with sampling weights: Is there a “best” approach?. Environ. Res..

[23] Pardeshi G.S. (2010). Age heaping and accuracy of age data collected during a community survey in the yavatmal district, maharashtra. Indian J. Community Med..

[24] Rader K.A., Lipsitz S.R., Fitzmaurice G.M., Harrington D.P., Parzen M., Sinha D. (2017). Bias-corrected estimates for logistic regression models for complex surveys with application to the United States’ Nationwide Inpatient Sample. Stat. Methods Med Res..

[25] Sun J., Luo H. (2017). Evaluation on equality and efficiency of health resources allocation and health services utilization in China. Int. J. Equity Health.

[26] Pappa E., Niakas D. (2006). Assessment of health care needs and utilization in a mixed public-private system: The case of the Athens area. BMC Health Serv. Res..

[27] Janković J., Simić S., Marinković J. (2010). Inequalities that hurt: Demographic, socio-economic and health status inequalities in the utilization of health services in Serbia. Eur. J. Public Health.

[28] Lu L., Zeng J., Zeng Z. (2017). What limits the utilization of health services among china labor force? analysis of inequalities in demographic, socio-economic and health status. Int. J. Equity Health.

[29] Qian Y., Zhou Z., Yan J., Gao J., Wang Y., Yang X., Xu Y., Li Y. (2017). An economy-ralated equity analysis of health service utilization by women in economically underdeveloped regions of western China. Int. J. Equity Health.

[30] Li C., Dou L., Wang H., Jing S., Yin A. (2017). Horizontal Inequity in Health Care Utilization among the Middle-Aged and Elderly in China. Int J. Environ. Res. Public Health.

[31] Wang L., Wang A., FitzGerald G., Si L., Jiang Q., Ye D. (2016). Who benefited from the New Rural Cooperative Medical System in China? A case study on Anhui Province. BMC Health Serv. Res..

[32] Guo Z., Guan X., Shi L. (2017). The impacts of implementation of National Essential Medicines Policies on primary healthcare institutions: A cross-sectional study in China. BMC Health Serv. Res..

[33] Zhang L., Liu S., Zhang G., Wu S. (2015). Internal migration and the health of the returned population: A nationally representative study of China. BMC Public Health.

[34] Cattell V. (2001). Poor people, poor places, and poor health: The mediating role of social networks and social capital. Soc. Sci. Med..

